# Identifying Weekly Trajectories of Pain Severity Using Daily Data From an mHealth Study: Cluster Analysis

**DOI:** 10.2196/48582

**Published:** 2024-07-19

**Authors:** Claire L Little, David M Schultz, Thomas House, William G Dixon, John McBeth

**Affiliations:** 1 Centre for Epidemiology Versus Arthritis University of Manchester Manchester United Kingdom; 2 Department of Earth and Environmental Sciences Centre for Atmospheric Science University of Manchester Manchester United Kingdom; 3 Centre for Crisis Studies and Mitigation University of Manchester Manchester United Kingdom; 4 Department of Mathematics University of Manchester Manchester United Kingdom; 5 NIHR Manchester Musculoskeletal Biomedical Research Unit Central Manchester University Hospitals NHS Foundation Trust Manchester United Kingdom; 6 School of Primary Care, Population Sciences and Medical Education University of Southamptom Southampton United Kingdom

**Keywords:** mobile health, mHealth, pain, cluster, trajectory, k-medoids, transition, forecast, mobile phone

## Abstract

**Background:**

People with chronic pain experience variability in their trajectories of pain severity. Previous studies have explored pain trajectories by clustering sparse data; however, to understand daily pain variability, there is a need to identify clusters of weekly trajectories using daily pain data. Between-week variability can be explored by quantifying the week-to-week movement between these clusters. We propose that future work can use clusters of pain severity in a forecasting model for short-term (eg, daily fluctuations) and longer-term (eg, weekly patterns) variability. Specifically, future work can use clusters of weekly trajectories to predict between-cluster movement and within-cluster variability in pain severity.

**Objective:**

This study aims to understand clusters of common weekly patterns as a first stage in developing a pain-forecasting model.

**Methods:**

Data from a population-based mobile health study were used to compile weekly pain trajectories (n=21,919) that were then clustered using a k-medoids algorithm. Sensitivity analyses tested the impact of assumptions related to the ordinal and longitudinal structure of the data. The characteristics of people within clusters were examined, and a transition analysis was conducted to understand the movement of people between consecutive weekly clusters.

**Results:**

Four clusters were identified representing trajectories of *no or low pain* (1714/21,919, 7.82%), *mild pain* (8246/21,919, 37.62%), *moderate pain* (8376/21,919, 38.21%), and *severe pain* (3583/21,919, 16.35%). Sensitivity analyses confirmed the 4-cluster solution, and the resulting clusters were similar to those in the main analysis, with at least 85% of the trajectories belonging to the same cluster as in the main analysis. Male participants spent longer (participant mean 7.9, 95% bootstrap CI 6%-9.9%) in the *no or low pain* cluster than female participants (participant mean 6.5, 95% bootstrap CI 5.7%-7.3%). Younger people (aged 17-24 y) spent longer (participant mean 28.3, 95% bootstrap CI 19.3%-38.5%) in the *severe pain* cluster than older people (aged 65-86 y; participant mean 9.8, 95% bootstrap CI 7.7%-12.3%). People with fibromyalgia (participant mean 31.5, 95% bootstrap CI 28.5%-34.4%) and neuropathic pain (participant mean 31.1, 95% bootstrap CI 27.3%-34.9%) spent longer in the *severe pain* cluster than those with other conditions, and people with rheumatoid arthritis spent longer (participant mean 7.8, 95% bootstrap CI 6.1%-9.6%) in the *no or low pain* cluster than those with other conditions. There were 12,267 pairs of consecutive weeks that contributed to the transition analysis. The empirical percentage remaining in the same cluster across consecutive weeks was 65.96% (8091/12,267). When movement between clusters occurred, the highest percentage of movement was to an adjacent cluster.

**Conclusions:**

The clusters of pain severity identified in this study provide a parsimonious description of the weekly experiences of people with chronic pain. These clusters could be used for future study of between-cluster movement and within-cluster variability to develop accurate and stakeholder-informed pain-forecasting tools.

## Introduction

### Background

Chronic pain (ie, pain lasting ≥3 months) is a common symptom of many long-term health conditions [1,2] and is associated with poor quality of life, poor health outcomes, and low participation in work and social activities [3,4]. There is substantial day-to-day variability in the severity of pain experienced [5,6], and people with chronic pain report that this variability leads to feelings of frustration and uncertainty about future pain [7,8]. Studies have identified associations between pain variability and response to treatment [9] as well as lower quality of life [10,11]. However, pain variability remains underestimated by researchers [12].

One way to explore pain variability is to cluster common pain trajectories and quantify movement between clusters. Previous studies have identified patterns of pain severity by clustering pain trajectories among individuals with chronic pain. These studies have often used sparse data on pain severity collected once per week [13], once per month [14], or less frequently [15]. These studies inform our understanding of longer-term experiences of chronic pain but not the day-to-day experience of pain severity that is important to patients. There is a need to extend this knowledge of pain clusters to within-week pain trajectories. Recent advances in mobile health (mHealth) methods that support the collection of data in the patients’ own environments [16,17], often using their own devices (eg, smartphones and tablets) [18], offer the opportunity to capture daily pain severity data.

It is also possible to explore movement between clusters of pain data; for example, Rahman et al [19] used changes between pain severity scores (not necessarily day-to-day changes) to identify 2 clusters of low pain volatility and high pain volatility. The authors then predicted movement between these clusters at 6-month intervals. However, there is a need to explore movement between clusters on a shorter time frame.

Once identified, weekly pain trajectories could be forecast. People living with chronic pain have reported that a pain forecast would reduce unpredictability and could be used to support planning daily activities, such as shopping, chores, and social participation [20,21]. In a research prioritization study, 75% of the respondents to a survey said they would use a pain forecast and prioritized a model that predicted daily fluctuations (ie, relatively short-term variability) and pain flares (patterns across multiple days) [20].

**Figure 1:**
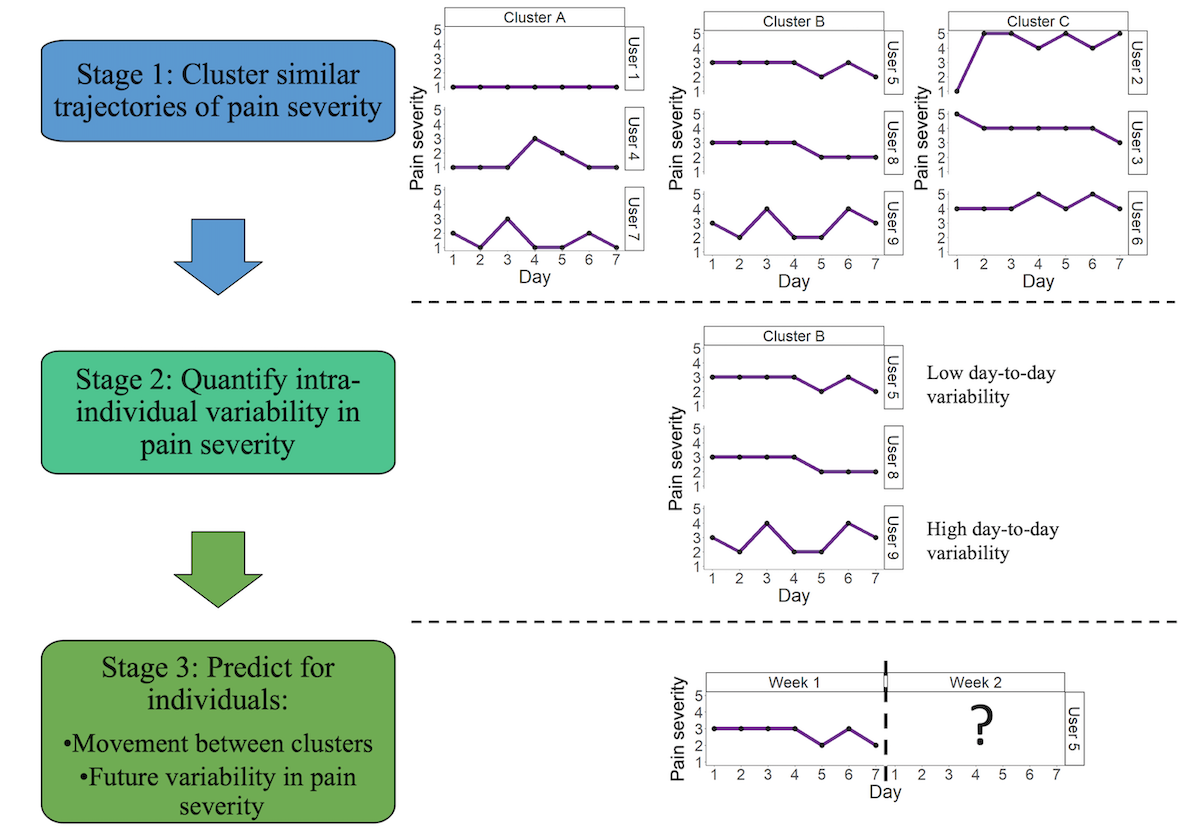
Three stages to build a pain forecast using data from a mobile health study. Data used in this figure are for illustrative purposes only (to provide an example of how data may be used in the pipeline of developing a pain forecast). First, data are clustered to identify common trajectories of weekly pain severity. Second, the remaining variability is explored for each trajectory within a cluster. The process is repeated for each cluster. Third, movement between clusters on consecutive weeks and the amount of day-to-day variability are predicted for an individual. The process is repeated for each individual.

We propose 3 stages to develop a pain forecast (Figure 1). Stage 1 identifies common weekly trajectories of pain severity using cluster analyses. Stage 2 investigates day-to-day variability in trajectories of pain severity for individuals within each cluster. These first 2 stages provide a better understanding of an individual’s pain experiences. Stage 3 predicts for an individual their movement between clusters of pain severity across consecutive weeks and future within-cluster day-to-day variability. This study focuses on the first of these stages: clustering trajectories of pain severity. Understanding clusters of weekly trajectories is an important stage in this forecasting model to identify group-level associations that may be masked by population-level analysis.

Once daily pain severity data are collected, there are several challenges to overcome in clustering these data for use in a pain-forecasting model. First, patient-generated health data are often collected on an ordinal scale. However, equal intervals between responses cannot be assumed, and using metric models to analyze ordinal data can lead to errors [22]. Second, data collected are longitudinal, and algorithms used for clustering should respect this longitudinal feature of the data. Third, clusters of pain severity that will be used in a pain-forecasting model should be interpretable to end users. To address these challenges, it is necessary to identify and use a suitable method for clustering patient-generated health data. Any assumptions made about the data should be tested in sensitivity analyses to ensure robustness. Observing substantial movement between clusters would suggest the feasibility of forecasting cluster movement in future work. Therefore, understanding the characteristics of individuals who contribute to different clusters and how these individuals move between clusters over time will aid end-user interpretability.

### Objectives

The aim of this study was to understand pain severity clusters in people living with chronic pain. The specific objectives were to (1) use a suitable algorithm to identify the optimum number of clusters of pain trajectories, (2) conduct sensitivity analyses to test assumptions made when clustering data, (3) examine the characteristics of people within clusters, and (4) describe the movement of people between different clusters over time.

## Methods

### Data Source

This study is a secondary analysis of a population-based mHealth study, Cloudy with a Chance of Pain [16,23,24]. Study participants were recruited between January 2016 and January 2017 through advertisements on television, radio, and social media. Data collection ended in April 2017, with participants able to contribute data for between 0 and 15 months. The inclusion criteria were as follows: participants with chronic pain, aged ≥17 years, living in the United Kingdom, and owning an Android or iOS smartphone. Participants downloaded a co-designed mobile phone app; gave electronic consent; and provided demographic information, including their sex (male or female), year of birth (entered as free text), and pain conditions (selected from a list of predefined responses, eg, rheumatoid arthritis and fibromyalgia). Daily reports of 10 variables were collected, including pain severity. Participants were asked “How severe was your pain today?” They responded by selecting *no pain* (score=1), *mild pain* (score=2), *moderate pain* (score=3), *severe pain* (score=4), or *very severe pain* (score=5). Daily reports of other variables included mood, fatigue, and physical activity, but these were not included in this secondary analysis. Data were collected locally on the smartphone, transferred to an external server where they were anonymized, and then returned to the researchers in anonymized form. Daily data could be contributed for a maximum of 15 months, with participants requested to track symptoms for 6 months. In total, 10,584 people downloaded the app and recorded their demographic information and at least 1 record of pain severity. Of the 10,584 participants, 8554 (80.82%) were female, with a mean age of 51 (SD 12.5) years. On average, these participants contributed pain severity data on 76 days (10,067/10,584, 95.12% of the participants contributed data on between 1 and 359 days). Previous analysis of these data classified participants as *highly engaged* (865/6370, 13.58%), *moderately engaged* (1384/6370, 21.73%), *less engaged* (2503/6370, 39.4%), and *tourists* (1618/6370, 25.4%) [24].

### Ethical Considerations

Ethics approval for the Cloudy with a Chance of Pain mHealth study was obtained from the University of Manchester Research Ethics Committee (ethics/15522) and from the National Health Service Integrated Research Application System (23/NW/0716). Participants were required to provide electronic consent for study inclusion. Anonymized data were received by the research team. Further ethics approval was not required for the secondary analysis described in this study.

### Data Preparation

**Figure 2:**
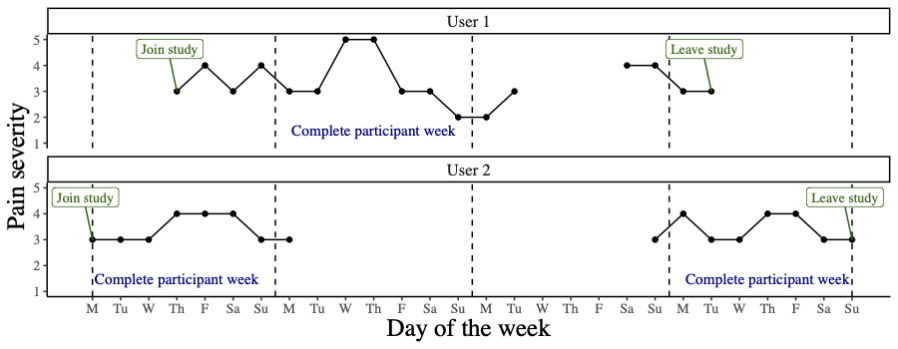
Example selections of complete trajectory weeks for 2 participants. The participants join and leave the study at different times. One complete participant week from user 1 is included in the analysis. Two complete participant weeks from user 2 are included in the analysis.

For this study, weekly trajectories of pain severity data were used. To align data across multiple respondents, trajectories beginning on a Monday were identified. This alignment introduced a structure to the data based on the work week to mitigate the impact of individuals entering the study at different times and to deal with day-of-the-week effects. A complete participant week was defined as complete pain severity data contributed by a single participant during a single calendar week (Monday-Sunday; Figure 2). Pain severity data from a complete participant week were included in the analysis if (1) the participant had joined the study on or before the Monday, (2) the participant had remained in the study on or after the following Sunday, and (3) the participant had provided complete pain severity data (ie, 1 pain severity score on each of the 7 days). Multiple complete participant weeks could be included in the analysis for each participant (up to 64 weeks due to the length of the study).

### Statistical Methods

#### Identifying the Optimal Number of Clusters

Previous studies have used a range of methods to cluster pain severity, including k-means clustering [25,26], hierarchical cluster analysis [27], growth mixture modeling [28-31], latent class growth analysis [13-15,32,33], multilevel latent class analysis [34], and group-based trajectory analysis [35-40]. Different approaches have different strengths and kinds of assumptions; for example, some may assume that clusters are internally homogeneous, while others may assume that the data overall follow a particular (eg, linear) form, are continuous, or are similar. In fact, clustering of ordinal and longitudinal data using a model that explicitly represents these features in a computationally inexpensive way remains a major unsolved methodological challenge. In this study, we chose a method that does not make strong assumptions about the form or generating mechanism of the data or the within-cluster variance and maintains the assumption that the data are ordinal in nature while having excellent computational performance and convergence properties. However, the assumption about the longitudinal nature of the data is relaxed.

To identify the optimal number of clusters, data were summarized in feature vectors, compared using the Manhattan (*ℓ*_1_) distance measure, and clustered using an adaptation of the k-medoids algorithm, detailed herein. The feature vectors were 7D, with entries representing the pain severity data on each of the 7 days in a complete participant week. Using the data directly in this way ensured that feature vectors remained interpretable. The differences between feature vectors were found by calculating the Manhattan distance through entry-wise summation of absolute differences to respect the ordinal nature of the outcome variable. The implementation of the k-medoids algorithm used to cluster the feature vectors can be derived as follows. A k-medoids algorithm randomly assigns user-defined k feature vectors to be the cluster centers (or medoids) and then iteratively (1) assigns each feature vector to the closest medoid and (2) recalculates the medoid of the clusters. The term *medoid* refers to the use of actual data points as the centers for the clusters [41]. Such use of observed data as centers for the clusters prevents outputs such as “pain severity of 3.2” that might arise if means are used and that are uninterpretable and erroneously assume an interval scale. To implement the k-medoids algorithm, the Clustering Large Applications (CLARA) program was used, which was specifically designed to be used with large data sets to reduce overall computation time [42,43].

A k-medoids algorithm requires a user-defined value for the number of clusters (k) in the data [41]. The implementation of the CLARA program was therefore repeated for values of k from 1 to 20. The output of the algorithm can be sensitive to the random feature vectors selected as the medoids in the first stage of the algorithm. The algorithm was therefore repeated 20 times, once for each value of k. At each iteration, the remaining variance within each cluster was calculated as the within-cluster sum of squares (WSS). The WSS calculates the total remaining distance between pairs of feature vectors in the same cluster. For each value of k, the iteration that returns the smallest value of WSS is selected and reported on a plot.

The optimal number of clusters was then selected using 3 criteria. First, from the plot of k against WSS, the optimal number of clusters was chosen visually using the elbow method [44]. While ideally a formal trade-off would be made between model complexity and goodness of fit, there is no clear method to use. Existing methods (eg, information criteria, silhouette method, and gap statistic) can suggest different numbers of clusters [45], possibly due to underpenalizing the complexity of data sets of the size used in this study. Therefore, the less formal elbow method allows us to be more explicit in the judgments we make to resolve the absence of an unambiguous method for learning cluster numbers from data. Second, clusters were required to contain 5% of the trajectories, similar to previous studies [13,36,46-49]. Third, cluster solutions were examined for clinical interpretability. For this measure, candidate solutions were examined to ensure meaningful differences between the cluster medoids. Furthermore, the distribution of the demographic data of participants contributing trajectories to each cluster were examined to ensure that the results reflected expected distributions.

#### Sensitivity Analyses

#### Overview

Three sensitivity analyses were conducted to test assumptions made in the main analysis, with the methodology behind choosing these being to modify assumptions made about the data and to see whether the broad conclusions were robust. Robust conclusions would be indicative of a strong model-independent signal in the data, even if modified assumptions led to a less interpretable output. The main analysis assumed that data were on an ordered scale but relaxed the assumption that the data were longitudinal.

#### Sensitivity Analysis 1

The first sensitivity analysis maintained the longitudinal nature of the data but implicitly assumed that the outcome variable was on a continuous scale. Feature vectors were compared using the Euclidean distance, which erroneously assumes regular intervals between values on the pain severity scale. However, the use of the Euclidean distance permits the use of the *KmL* package, which specifically clusters longitudinal data [50]. The *KmL* package is an adaptation of the k-means algorithm. The k-means algorithm is similar to k-medoids, but the center of each cluster is calculated using the mean of the feature vectors assigned to the cluster. The use of the *KmL* package, instead of the CLARA program, and the resulting use of mean trajectories rather than medoid trajectories were the only adaptions to this sensitivity analysis. The feature vectors, the 20 repetitions of the algorithm for each value of k, and the use of the elbow method to select k remained unchanged.

#### Sensitivity Analysis 2

The second sensitivity analysis relaxed assumptions about the longitudinal nature as well as the ordinal nature of the outcome variable. In this sensitivity analysis, the data were not assumed to be longitudinal, and the outcome variable was assumed to be unordered categorical data. A different feature vector was used that converted ordinal pain severity values into dummy variables using one-hot encoding. In this encoding, there were 35 binary categories, each representing a unique day and pain severity category. The feature was recorded as *1* if the pain severity score was seen on that day and *0* otherwise. In this way, 7 of the features were recorded as *1* for each complete participant week. The feature vectors were compared using the Jaccard distance, typically used for such vectors of binary data. The cluster analysis was then conducted using the CLARA program in the same manner as described in the main analysis.

#### Sensitivity Analysis 3

The third sensitivity analysis challenged the definition of a Monday-to-Sunday week when defining complete participant weeks. Instead, the following analysis was conducted for each day (*D*) in the week. Complete participant weeks were selected from the original data for each participant when there were pain severity data for each day in the *D*-to-*D*+6 week (eg, Wednesday-Tuesday week). On each new data set corresponding to a different *D*, clustering was conducted using the CLARA program at each value of k between 1 and 20, as described in the main analysis. Due to the adapted complete participant weeks, individuals may have contributed different numbers of weeks to the sensitivity analysis.

#### Calculations for Each Sensitivity Analysis

For each sensitivity analysis, the optimal number of clusters was calculated. Similar numbers and descriptions of the clusters would provide evidence that the conclusions from the main analysis are robust. Furthermore, for each cluster in the main analysis, the proportion of trajectories allocated to the same cluster in each sensitivity analysis was calculated. A high proportion would further suggest that the results are robust to the assumptions made by using the Manhattan distance and k-medoids algorithm in the main analysis.

### Description of Clusters

Information about the trajectories assigned to each of the clusters in the optimal solution was summarized. First, the number of trajectories assigned to each cluster was reported. Second, the clusters were visualized with a spaghetti plot of individual trajectories and the medoid of each cluster. Finally, the average proportion of time spent in each cluster by each participant was calculated. This information was summarized by calculating the mean proportion of time spent in each cluster across demographics (ie, age, sex, and chronic pain condition or conditions).

### Transition Between Clusters

**Figure 3:**
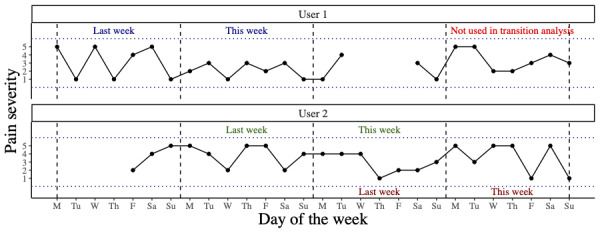
Example data from 2 participants highlighting how their data are used to examine transitions between clusters. User 1 provided data in 3 complete participant weeks. The first 2 are consecutive and therefore are used in the transition analysis. The final complete participant week is not used. User 2 provided 3 complete participant weeks. All three are used in the transition analysis. The middle week is labeled as both this week and last week in different pairings.

For the optimal solution of clusters in the main analysis, the transition of individuals between clusters on consecutive weeks was examined. To do this, a subset of the total data was used. Complete participant weeks (*this week*) were retained if the participant had also contributed a complete participant week in the directly preceding week (*last week*). A trajectory could be labeled as both *this week* and *last week* if there were both preceding and succeeding weeks for the individual (Figure 3). The demographic data of participants included in this transition analysis were compared to those included in the main analysis.

Each trajectory was assigned a cluster in the CLARA program of the k-medoids cluster algorithm. The transition probabilities were then calculated as follows. For all trajectories in each cluster *last week*, the percentage of trajectories that transitioned to each cluster *this week* were calculated. The resulting percentages are reported in a transition matrix.

Data were analyzed in R (version 4.1.2; R Foundation for Statistical Computing). The reporting of the analysis followed the STROBE (Strengthening the Reporting of Observational Studies in Epidemiology) guidelines [51].

## Results

### Data Source

There were 2807 participants who contributed 21,919 participant weeks of data to this analysis. The participants’ mean age was 51.2 (SD 12.8) years, and 83.11% (2333/2807) were female. Table 1 reports the number of participants by age, sex, chronic pain condition, and the average number of participant weeks contributed to the analysis by members of the subgroup. Overall, older participants contributed a greater number of participant weeks than younger participants. Male participants contributed slightly more (8.1) participant weeks than female participants (7.7). Participants with osteoarthritis (9.1) and unspecific arthritis (9.0) contributed the highest number of participant weeks, and participants with chronic headache (6.0) contributed the fewest participant weeks. Comorbid conditions are described in Tables S1 and S2 in Multimedia Appendix 1.

**Table 1:** Demographic information of the participants who contributed to the analysis and the average number of participant weeks contributed by each subgroup (n=2807).

Demographic information			Participants, n (%)		Weekly trajectories contributed by participants, mean (SD)
**Age group (y)**					
	17-24	67 (2.39)		5.2 (8.6)	
	25-34	255 (9.08)		5.6 (7.1)	
	35-44	508 (18.1)		6.8 (9.0)	
	45-54	755 (26.9)		7.5 (9.1)	
	55-64	788 (28.07)		8.6 (9.7)	
	65-86	434 (15.46)		9.9 (10.5)	
**Sex**					
	Female	2333 (83.11)		7.7 (9.4)	
	Male	474 (16.89)		8.1 (9.4)	
**Chronic pain condition^a^**					
	Rheumatoid arthritis	548 (19.52)		7.7 (8.6)	
	Osteoarthritis	975 (34.73)		9.1 (10.3)	
	Spondyloarthropathy	254 (9.05)		7.6 (8.6)	
	Gout	96 (3.42)		7.8 (10.6)	
	Unspecific arthritis	1028 (36.62)		9.0 (10.3)	
	Fibromyalgia	718 (25.58)		7.1 (8.9)	
	Chronic headache	274 (9.76)		6.0 (6.5)	
	Neuropathic pain	427 (15.21)		7.5 (9.7)	
	Other or no medical diagnosis	668 (23.8)		6.9 (8.9)	

^a^Percentages exceed 100% because participants could report multiple chronic pain conditions.

### Identifying the Optimal Number of Clusters

**Figure 4:**
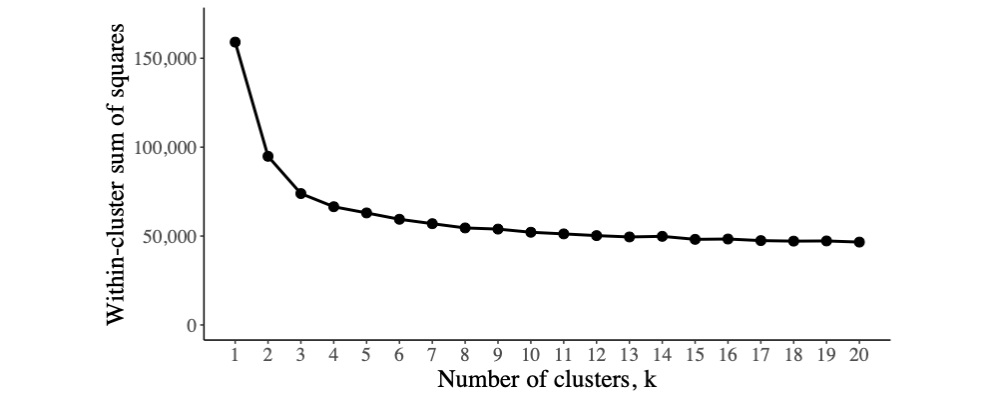
Unexplained variability across different cluster (k) solutions. The within-cluster sum of squares indicates the remaining variance within clusters. An elbow at k=4 suggests an appropriate solution, with diminishing returns for the inclusion of further clusters.

The results of the CLARA algorithm are shown herein. Figure 4 reports the remaining variability within clusters as the WSS at each value of k. There is an elbow at k=4, suggesting that most of the observed variability can be explained by a solution with 4 clusters, with diminishing returns for including further clusters in the solution. Four clusters reduced the WSS from 159,100 to 66,507; therefore, the clustering algorithm describes 58.2% of the variability in the data. Each cluster contained >5% of the pain trajectories. Therefore, 4 clusters provide an appropriate choice for these data.

**Figure 5:**
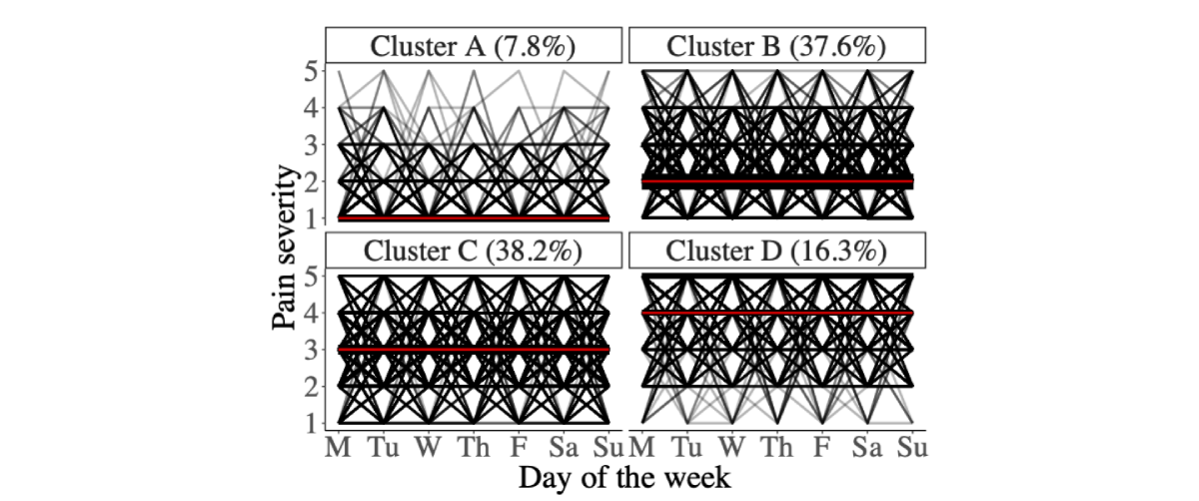
Weighted spaghetti plot of trajectories assigned to each cluster. The weight (and transparency) of each path represents the number of trajectories following that path. The red line represents the medoid of the cluster. Cluster A=no or low pain, cluster B=mild pain, cluster C=moderate pain, and cluster D=severe pain. The percentage of trajectories assigned to each cluster is shown.

The trajectories in each cluster are shown in the spaghetti plot in Figure 5. Trajectories are weighted such that thicker lines represent a higher number of trajectories following the path. The red line represents the medoid of the k-medoids algorithm. The clusters can be named by examining the medoid: A=*no or low pain*, B=*mild pain*, C=*moderate pain*, and D=*severe pain*. Of the 21,919 trajectories, cluster A contained 1714 (7.82%), cluster B contained 8246 (37.62%), cluster C contained 8376 (38.2%), and cluster D contained 3583 (16.35%).

### Sensitivity Analyses

#### Sensitivity Analysis 1 (KmL Algorithm and Euclidean Distance)

**Figure 6:**
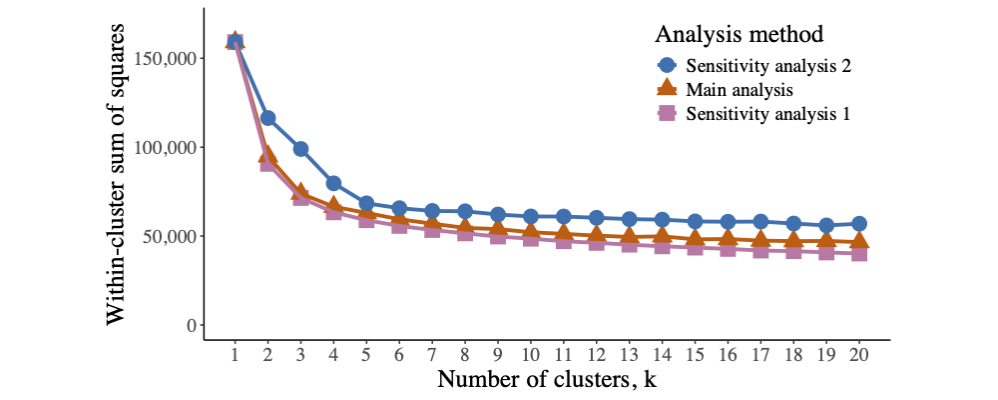
Unexplained variability across different cluster (k) solutions for the main analysis and 2 sensitivity analyses. In the main analysis and sensitivity analysis 1, there is an elbow at k=4. In sensitivity analysis 2, there is an elbow at k=5.

Full results are presented in Multimedia Appendix 2. The plot visualizing WSS against k for this analysis is similar to that of the main analysis and has an elbow at k=4 (Figure 6). The optimal 4-cluster solution describes 60% of the observed variability. The descriptions of the spaghetti plots (ie, cluster A=*no or low pain*, cluster B=mild pain, cluster C=*moderate pain*, and cluster D=*severe pain* ) are the same as those in the main analysis, despite the use of a mean rather than a medoid to describe the average trajectory in each cluster. Of the 21,919 trajectories, 18,895 (86.2%) were assigned to the same cluster as in the main analysis, indicating similar results. Clusters B and C remain the largest clusters (8484/21,919, 38.71% and 8001/21,919, 36.5% trajectories, respectively), although cluster A is larger in this sensitivity analysis than in the main analysis (2493/21,919, 11.37% vs 1714/21,919, 7.82%).

#### Sensitivity Analysis 2 (CLARA Algorithm and Jaccard Distance)

Full results are presented in Multimedia Appendix 2. The plot of k against WSS for this analysis has an elbow at k=5 (Figure 6). However, 1 cluster contained 990 (4.52%) of the 21,919 trajectories in the 5-cluster solution, which did not meet the criteria for cluster sizes >5%, and therefore a 4-cluster solution remained optimal in this analysis. A 4-cluster solution describes 50% of the variability. Spaghetti plots of the 4-cluster solution show the same descriptions as those in the main analysis. In total, 20,197 (92.14%) of the 21,919 trajectories were assigned to the same cluster as in the main analysis.

#### Sensitivity Analysis 3 (Day of the Week)

Full results are presented in Multimedia Appendix 3. Each plot of WSS against k suggested an optimal solution at k=4. The proportions of trajectories assigned to each cluster in each 4-cluster solution are similar to those in the main analysis. The proportions in cluster A ranged between 7.72% (1717/22,255) and 7.9% (1769/22,404), cluster B between 37.49% (8273/22,067) and 37.71% (8393/22,255), cluster C between 38.04% (8523/22,404) and 38.43% (8481/22,067), and cluster D between 16.19% (3614/22,320) and 16.42% (3679/22,404). These results show that the main analysis is robust to the day of the week on which the trajectories begin.

### Description of Clusters

The average proportions of time spent in different clusters across different characteristics (age, sex, and condition) are summarized in Table 2. The participants in the oldest age bracket (65-86 y) spent less time (mean 9.8, 95% bootstrap CI 7.7%-12.2%) in the *severe pain* cluster than those in the youngest age bracket (17-24 y; mean 28.3, 95% bootstrap CI 19%-38%). Female participants spent more time in the *severe pain* cluster (mean 18, 95% bootstrap CI 16.6%-19.3%) than male participants (mean 12.3, 95% bootstrap CI 10%-14.7%) and less time in the *lowest pain* cluster (female participants: mean 6.5, 95% bootstrap CI 5.8%-7.3%); male participants: mean 7.9, 95% bootstrap CI 6.1%-10%). Participants with fibromyalgia and neuropathic pain spent the most time in the *severe pain* cluster (mean 31.5, 95% bootstrap CI 28.8%-34.5%) and (mean 31.1, 95% bootstrap CI 27.2%-35.1%, respectively). Participants with rheumatoid arthritis spent the most time in the *lowest pain* cluster (mean 7.8, 95% bootstrap CI 6%-9.6%).

**Table 2:** Percentage of time spent in each cluster by baseline characteristic (for each characteristic, the average percentage of time spent in each cluster by members of the characteristic is reported).

		Time spent in cluster A (%), mean (95% CI)	Time spent in cluster B (%), mean (95% CI)	Time spent in cluster C (%), mean (95% CI)	Time spent in cluster D (%), mean (95% CI)
All		6.7 (6.0-7.5)	36.8 (35.4-38.2)	39.4 (38.0-40.9)	17.0 (15.9-18.2)
**Age group (y)**					
	1­7-24	3.4 (0.8-6.9)	28.5 (19.9-38.1)	39.8 (30.6-49.3)	28.3 (19.0-38.0)
	25-34	6.7 (4.3-9.4)	32.0 (27.6-36.5)	41.5 (36.9-46.4)	19.8 (15.5-24.2)
	35-44	5.3 (3.9-6.9)	31.5 (28.4-34.6)	38.6 (35.2-42.1)	24.5 (21.2-27.9)
	45-54	5.6 (4.5-6.9)	36.3 (33.6-39.0)	39.4 (36.7-42.1)	18.7 (16.5-21.0)
	55-64	8.4 (6.8-10.1)	38.5 (35.9-41.0)	40.4 (37.9-43.1)	12.7 (10.9-14.7)
	65-86	7.9 (6.1-9.9)	44.9 (41.6-48.4)	37.3 (33.9-40.6)	9.8 (7.7-12.2)
**Sex**					
	Female	6.5 (5.8-7.3)	35.6 (34.1-37.2)	39.9 (38.4-41.4)	18.0 (16.6-19.3)
	Male	7.9 (6.1-10.0)	42.5 (39.1-46.1)	37.2 (34.0-40.5)	12.3 (10.0-14.7)
**Chronic pain condition^a^**					
	Rheumatoid arthritis	7.8 (6.0-9.6)	38.9 (35.7-42.0)	39.5 (36.5-42.5)	13.8 (11.5-16.2)
	Osteoarthritis	5.4 (4.4-6.5)	34.7 (32.5-37.0)	42.6 (40.2-44.9)	17.2 (15.2-19.3)
	Spondyloarthropathy	4.1 (2.5-6.1)	31.8 (27.5-36.2)	43.5 (38.9-47.9)	20.6 (16.3-25.0)
	Gout	6.1 (2.4-10.4)	33.0 (25.5-40.1)	41.6 (34.2-48.9)	19.3 (12.9-26.2)
	Unspecific arthritis	6.3 (5.2-7.5)	39.0 (36.6-41.3)	38.5 (36.3-40.7)	16.2 (14.3-18.2)
	Fibromyalgia	1.7 (1.0-2.4)	19.1 (17.0-21.3)	47.7 (45.0-50.5)	31.5 (28.8-34.5)
	Chronic headache	5.1 (3.1-7.4)	28.7 (24.6-33.2)	40.3 (35.9-44.7)	25.9 (21.5-30.4)
	Neuropathic pain	3.3 (2.0-4.7)	23.6 (20.4-27.0)	42.0 (38.4-45.8)	31.1 (27.2-35.1)
	Other or no medical diagnosis	6.7 (6.0-7.5)	36.8 (35.4-38.2)	39.4 (38.0-40.8)	17.0 (15.8-18.3)

^a^Percentages exceed 100% because participants could report multiple chronic pain conditions.

### Transition Between Clusters

There were 12,267 pairs of participant weeks from 1761 participants used in the transition analysis. The demographic data are compared to those in the main analysis in Multimedia Appendix 4. In general, a slightly higher proportion of older adults contributed to the transition analysis compared to the cluster analysis; for example, of the 2807 participants in the main analysis, 434 (15.46%) were aged 65 to 86 years, but of the 1761 participants in the transition analysis, 300 (17.04%) were older adults. There are no other differences in the demographics of participants contributing to the main analysis and the transition analysis.

**Figure 7:**
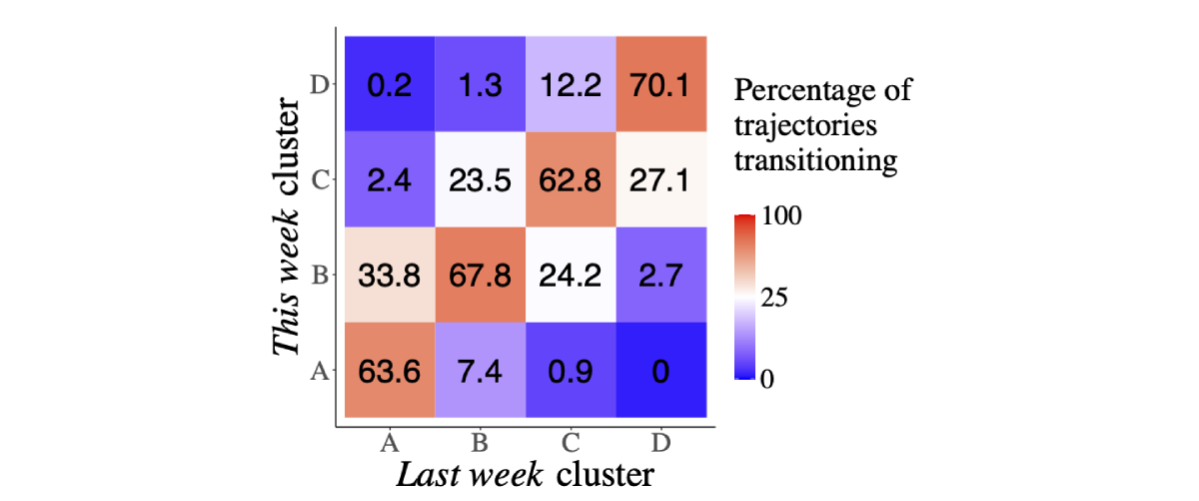
Transition matrix of movement between clusters on consecutive weeks. For membership in each cluster for last week, the percentage of membership in each cluster for this week is shown. Random movement between clusters would suggest that each combination has a transition percentage of 25%. Blue squares represent transitions that have a higher-than-random percentage (>25%). Red squares represent transitions that have a lower-than-random percentage (<25%). A white square would have exactly the random percentage (25%).

The percentages of consecutive trajectories transitioning between clusters are shown in Figure 7. For each cluster, the highest percentages of trajectories in consecutive weeks remain in the same cluster, with the percentage values ranging between 62.76% (2948/4697) and 70.14% (1466/2090). On average, 65.96% (8091/12,267) of the trajectories remain in the same cluster. When individuals move between clusters, it is most frequently to an adjacent cluster. There is a very small percentage of consecutive weeks displaying movement between clusters ≥2 levels away.

## Discussion

### Principal Findings

This study identified and described clusters of weekly trajectories of pain severity in a large population-based mHealth study to address 4 objectives in turn. First, we reported that 4 clusters (A=*no or low pain*, B=*mild pain*, C=*moderate pain*, and D=*severe pain*) represented an optimal clustering solution for these data. In this solution, clusters B and C contained the greatest number of weekly pain trajectories.

Second, we conducted sensitivity analyses to identify whether the conclusions made about the first objective were robust to modified assumptions around the structure of the data. Two sensitivity analyses were conducted when the outcome variable was assumed to be (1) continuous and longitudinal and (2) categorical and not longitudinal. These analyses found that 4 clusters remained a suitable conclusion. A third sensitivity analysis found no differences in the clusters of trajectories starting on different days of the week.

Third, younger people and female participants contributed a greater number of trajectories to the *severe pain* cluster than older people and male participants, respectively. Participants with fibromyalgia and neuropathic pain contributed more trajectories to the *severe pain* cluster than those with other pain conditions, whereas participants with rheumatoid arthritis contributed more trajectories to the *no or low pain* cluster than those with other pain conditions.

Fourth, we examined transitions between clusters and found that 65.96% (8091/12,267) of the consecutive trajectories contributed to the same cluster. However, there was clear evidence of between-cluster movement with 34.04% (4176/12,267) of the consecutive trajectories assigned to different clusters. Between-cluster movement was most likely to a neighboring cluster; for example, moving from cluster 1 to cluster 2 was more common than moving from cluster 1 to cluster 3. This analysis demonstrates that overall, individuals tend to experience similar patterns of pain severity from week to week, although there are substantial experiences of increases or decreases in pain severity, thereby reflecting the lived experience of people with chronic pain having variability in symptoms and noting how pain can fluctuate between weeks.

People with chronic pain have highlighted a need to describe and predict the variability in the severity of their pain. Through clustering, this study has described 4 common experiences of pain severity, accounting for two-thirds of the observed variability. However, trajectories within each cluster are not homogeneous, and there remains within-cluster variation. To better describe the individual weekly pain experience, future work should explore the remaining variability within clusters.

### Comparison With Prior Work

Many studies have identified clusters of pain trajectories among individuals living with chronic pain. Some have focused on participants with 1 chronic pain condition, such as osteoarthritis [15,36-39,52-58], low back pain [13,14,27,59-65], other back pain [25,32,49,66], neck or shoulder pain [33,61,67,68], leg pain [29], knee pain [69], or foot pain [70], whereas others have identified clusters among a broader population, such as those with musculoskeletal pain [26,31,47,71,72] or general pain [48,73-75]. Clusters in these studies were described by the severity of pain (eg, *no pain*, *very low pain*, *mild pain*, *moderate pain*, *high pain*, and *severe pain*), the level of change in pain severity (eg, *persistent*, *ongoing*, *episodic*, *worsening*, *recovering*, and *fluctuating*), or a combination of these features.

These previous studies have often considered only sparse data, with relatively large time intervals between consecutive data points. Of those gathering data for at least 1 year (n=27 studies), data were collected >2 times in only 2 studies [13,67]. In these 2 studies, data were collected weekly for 1 year to explore the course of specific pain conditions (neck pain and low back pain). Kongsted et al [13] used 12 models to identify between 5 and 12 clusters in each model. Clusters were described by the severity of pain (eg, *moderate* and *severe*) and also by the temporal features of the trajectories (eg, *episodic*, *recovery*, and *ongoing*). Pico-Espinosa et al [67] identified 6 clusters of pain described as *small improvement*, *moderate improvement*, *persistent*, *large improvement*, *slightly fluctuating*, and *highly fluctuating*. The clusters identified in our analysis were described by the severity of pain, similar to clusters in studies with sparse data. Our clusters were unlikely to identify long-term disease development, as with trajectories over longer periods.

Similar to our study, some previous studies have used methods from the k-means and k-medoids family of algorithms. Knecht et al [25] used the *KmL* package to identify 2 clusters in *responders* and *nonresponders* groups. Weng et al [76] used a k-median algorithm to identify 4 clusters of pain severity: *slightly rise*, *completely drop*, *sudden rise*, and *steady group*. Both these studies identified trajectories with changing pain severity, while our study identified trajectories of weekly pain where the medoid was stable across the week.

In all aforementioned studies, the experiences of individuals were described by a single trajectory across the full duration of follow-up, whereas our study examined week-to-week transition between clusters. Kongsted et al [77] have previously examined week-to-week pain severity across a year, using predefined clusters. The authors identified that 41% and 21% of the respondents in 2 different data sets had stable pain over a year, defined as pain within 1 point of the mean pain value on an 11-point numerical rating scale. The remaining pain trajectories were classified as having a single episode of pain, being *episodic* or *fluctuating*. The transitions identified in our study suggest stability between 65.96% (8091/12,267) of the consecutive weeks. However, some individuals in our study may experience the other longer-term descriptions outlined by Kongsted et al [77]; for example, an individual might not transition out of a cluster for most of the year; yet they might experience only 1 episode. Future studies should further examine the movement between different pain states and identify the drivers of these transitions.

### Strengths and Limitations

A number of strengths and limitations of this study should be considered. First, a strength was that participants could contribute daily data for up to 64 weeks. This frequent and granular data collection, enabled by mHealth, overcame limitations of sparse data collection in previous studies (as identified by Beukenhorst et al [78]). As a result, this study was able to analyze the weekly trajectories contributed by participants, determining common pain patterns among a population with chronic pain at a more granular scale than previously investigated.

Second, the analysis presented in this paper modeled weekly pain trajectories rather than individual people. In contrast to studies that assign each individual to a single cluster across the whole follow-up, individuals were able to transition between different pain clusters over time as their pain experience changed, and their condition developed. These transitions were observed in 34.04% (4176/12,267) of the consecutive weeks, and this flexibility can be used in future work to explore the mechanisms driving movement between clusters.

Third, assumptions about the ordinal and longitudinal form of the data were modified in sensitivity analyses. A 4-cluster solution was most suitable for each analysis, indicating a strong model-independent signal in the data and a more robust conclusion regarding the most suitable number of clusters. Furthermore, the assignment of trajectories to each cluster were similar in each analysis (at least 86% similarity), indicating further stability in the results. There were benefits to the use of both the k-medoids algorithm and the longitudinal adaptation of the k-means algorithm used in this analysis. First, neither of these methods requires parametric assumptions about the form of the data [50]. Second, no prior assumptions, including the shape of the trajectory, are required by the algorithms [79]. Therefore, this data-driven approach made limited assumptions about the form of the data.

There were also limitations to the study. The data used in this study were from a population-based study that represented the UK population. Cloudy with a Chance of Pain recruited participants from all UK postcodes, although male participants and those in the age brackets 17 to 34 years and ≥75 years were underrepresented in the study population [24]. Despite being a smaller population, older people and male participants contributed more trajectories on average and were more likely to contribute trajectories to a *less severe pain* category. As these clusters will be used in the development of a pain-forecasting model, clusters should be generalizable to the population with chronic pain, and there remains the possibility that different pain clusters and between-cluster transitions could be realized among those who contributed to the study did and those who did not. Although it is unlikely that our large study population would display pain clusters and transitions different from those of the population with chronic pain that would use a smartphone tracking app, it remains a possibility that should be explored in future studies.

This analysis further selected participants by the requirement to provide a week of complete pain severity data, thus excluding missing data. There are reasons that data might be missing not at random, including missing due to severe pain, missing due to low pain severity, and missing due to stable pain, that result in repetitive score input and thus disengagement. The transition analysis also further selected participants by requiring 2 weeks of complete pain severity data. However, the age, sex, and chronic pain conditions of respondents in the main analysis and transition analysis (Multimedia Appendix 4) were similar to those in the full-study population (see the first supplementary in the study by Dixon et al [16]), suggesting that the included participants were representative of the study population.

There were limitations in the method used for clustering. First, the absence of parametric assumptions in either the k-medoids algorithm or the *KmL* package resulted in goodness-of-fit measures being inappropriate [79]. Therefore, the elbow method was used to select the optimum number of clusters. However, the use of the elbow method introduces subjectivity. Second, both the k-medoids algorithm and the *KmL* package require random starting values for the cluster centers, which can add volatility to the results. This volatility was mitigated by repeating the algorithms 20 times each and selecting the solution with the lowest remaining variability within clusters.

### Conclusions

Previous research has highlighted a need to better understand pain variability experienced by individuals with chronic pain [20]. Feelings of uncertainty among people with chronic pain have led them to want to better understand the pain that they may experience in the future. Clustering weekly pain trajectories offers a first step to better understanding common experiences of pain severity. Once these common experiences are better described, they can be used in future work to predict movement between clusters.

There are limited methods available for clustering pain severity that respect the ordinal and longitudinal nature of patient-generated health data in a computationally inexpensive manner. The clustering method and subsequent sensitivity analyses presented in this paper suggest that the use of k-medoids is robust to assumptions about the data structure.

This study identified 4 distinct patterns of weekly pain severity: *no or low pain*, *mild pain*, *moderate pain*, and *severe pain*. These can be used to describe the short-term pain experiences of people with chronic pain. Future work is required to identify how these clusters can be used in a pain-forecasting model. First, there remains individual variability within clusters of pain severity. Participants in patient and public involvement studies have identified that fluctuations in pain severity should be forecast, and therefore within-cluster variability should be quantified to further understand the weekly pain experience of individuals. Second, the transition of individuals between clusters should be explored to identify the drivers of movement between pain clusters on an individual level. The clusters identified in this study and in future work to understand within-cluster variability and the drivers of movement between clusters will enable a future pain-forecasting model.

## Appendix

Multimedia Appendix 1 Comorbid pain conditions.

Multimedia Appendix 2 Sensitivity analyses 1 and 2.

Multimedia Appendix 3 Sensitivity analysis 3.

Multimedia Appendix 4 Demographic data of participants included in transition analysis.

## Abbreviations

CLARA: Clustering Large Applications
mHealth: mobile health
STROBE: Strengthening the Reporting of Observational Studies in Epidemiology
WSS: within-cluster sum of squares

## Data Availability

The data sets generated and analyzed during this study are available from the corresponding author on reasonable request. Data management and analyses were performed in R (version 4.1.2; R Foundation for Statistical Computing). The code is available from the corresponding author on reasonable request.
