# Supplementary material for: Identifying Weekly Trajectories of Pain Severity Using Daily Data From an mHealth Study: Cluster Analysis
Source: JMIR Mhealth Uhealth. 2024 Jul 19;12:e48582. doi: 10.2196/48582 (PMC11297369; doi:10.2196/48582)
Supplement: Multimedia Appendix 1 [file mhealth_v12i1e48582_app1.docx]

Table S1: Reported co-morbid pain conditions by condition

| Condition | Participants reporting condition: *n* | Participants reporting co-morbid pain condition:  *n (%)* |
| --- | --- | --- |
| Rheumatoid arthritis | 548 | 299 (54.6) |
| Osteoarthritis | 975 | 720 (73.8) |
| Spondyloarthropathy | 254 | 196 (77.2) |
| Gout | 96 | 91 (94.8) |
| Unspecific arthritis | 1028 | 688 (66.9) |
| Fibromyalgia | 718 | 577 (80.4) |
| Chronic headache | 274 | 237 (86.5) |
| Neuropathic pain | 427 | 379 (88.8) |
| Other/no medical diagnosis | 667 | 381 (57.1) |

Table S2: Most commonly reported co-morbid conditions

| Conditions | Participants reporting co-morbid pain conditions: *n* |
| --- | --- |
| Osteoarthritis and Unspecified Arthritis | 386 |
| Osteoarthritis and Fibromyalgia | 210 |
| Unspecified Arthritis and Fibromyalgia | 206 |
| Fibromyalgia and Neuropathic Pain | 193 |
| Fibromyalgia and Other Pain Condition | 187 |
| Osteoarthritis and Neuropathic Pain | 154 |
| Rheumatoid Arthritis and Osteoarthritis | 150 |
| Unspecified Arthritis and Neuropathic Pain | 144 |
| Osteoarthritis and Other Pain Condition | 133 |
| Unspecified Arthritis and Other Pain Condition | 123 |

The ten most common combinations of comorbid conditions are presented with the number of people reporting both conditions
