# Supplementary material for: Identifying Weekly Trajectories of Pain Severity Using Daily Data From an mHealth Study: Cluster Analysis
Source: JMIR Mhealth Uhealth. 2024 Jul 19;12:e48582. doi: 10.2196/48582 (PMC11297369; doi:10.2196/48582)
Supplement: Multimedia Appendix 2 [file mhealth_v12i1e48582_app2.docx]

# Supplementary Materials 2 (Sensitivity Analyses 1 & 2)

This section reports the results from two of the sensitivity analyses. In the main analysis, trajectories were compared using the Manhattan distance and clustered using the *k*-medoids algorithm. In the first sensitivity analysis, trajectories were compared using the Euclidean distance and clustered using the KmL package in R. In the second sensitivity analysis, trajectories were summarised in a one-hot encoded feature vector, compared using the Jaccard distance and clustered using the k-medoids algorithm.

For each of the sensitivity analyses, the methods of the main analysis are repeated. The results of these analyses are presented alongside the results for the main analysis.

## Identifying optimal number of clusters


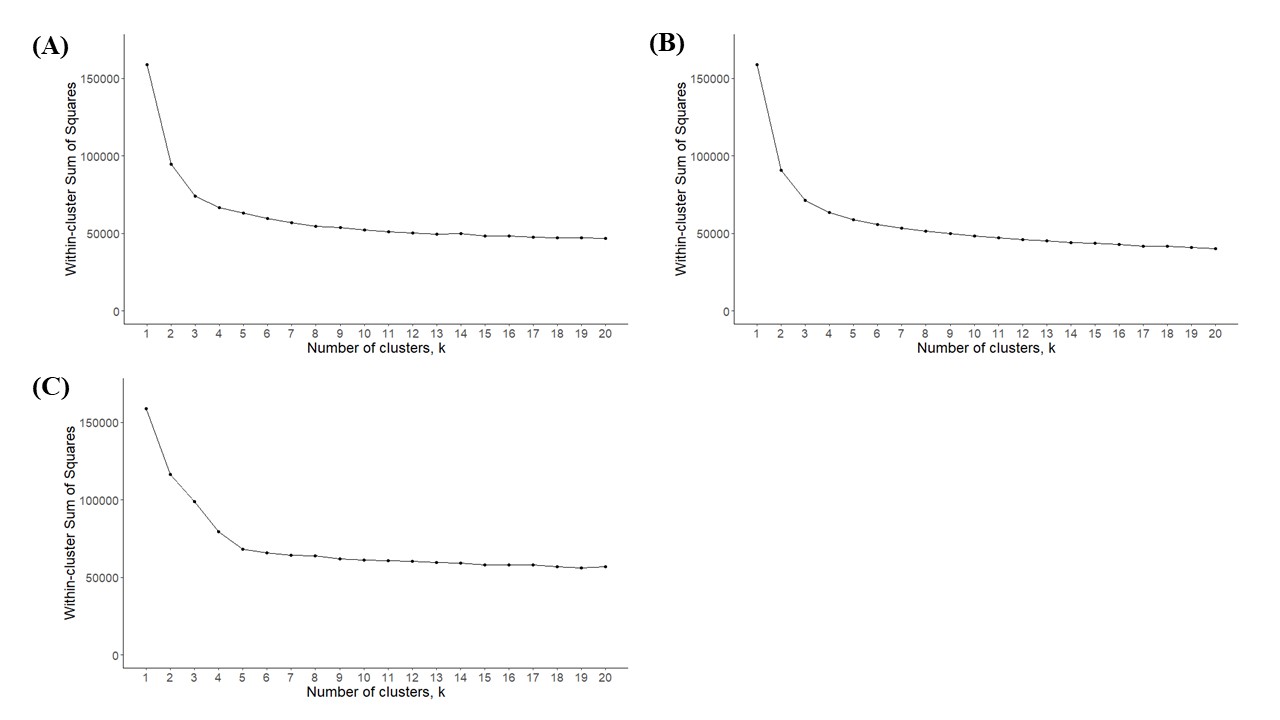
First, remaining variability within clusters (within-cluster sum of squares) was calculated for *k* (number of clusters) between 1 and 20. The results are presented in figure S1. There is an elbow at *k* = 4 for the first sensitivity analysis, with a four-cluster solution describing 60.0% of the observed variability. There is an elbow at *k* = 5 for the second sensitivity analysis, with a five-cluster solution describing 57.0% of the variability. In the following sections, both a four-cluster solution and five-cluster solution are explored for the second sensitivity analysis.

Figure S1: Unexplained variability among different cluster (k) solutions for (A) the main analysis, (B) the first sensitivity analysis and (C) the second sensitivity analysis.

## Description of clusters


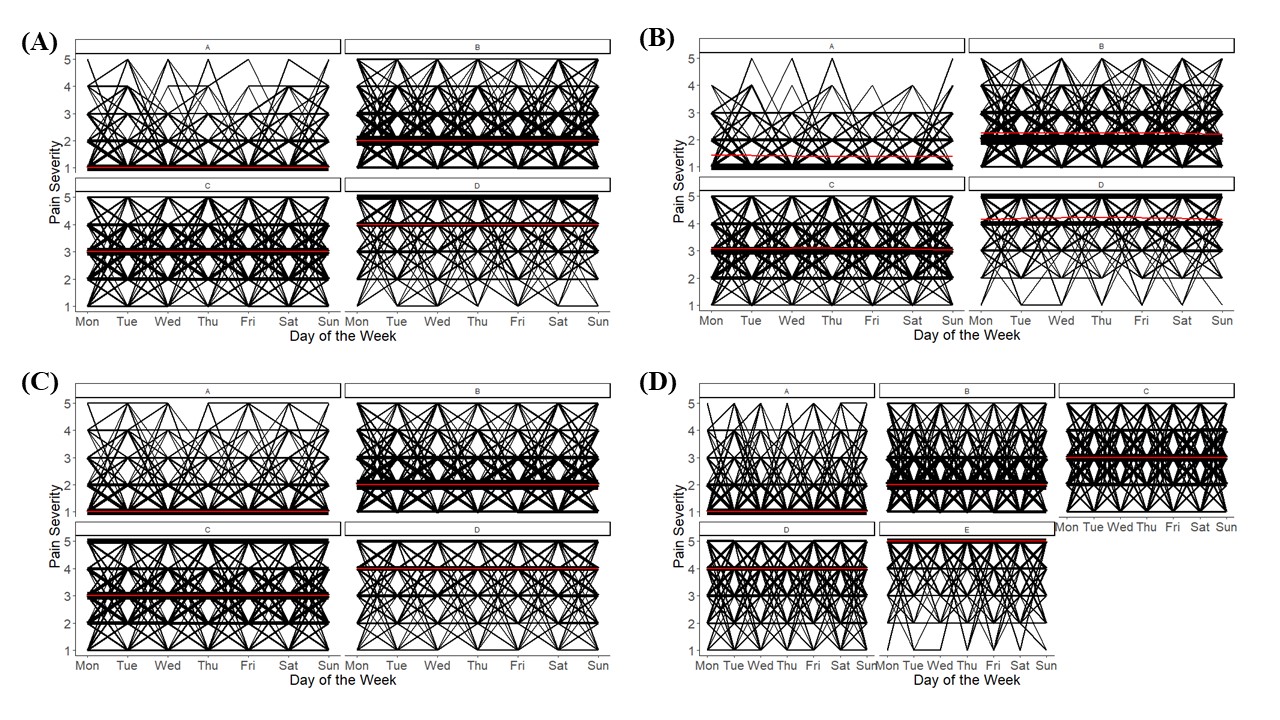
Four solutions are described in detail. These are: the four-cluster solution from the main analysis, the four-cluster solution from the first sensitivity analysis, the four-cluster solution from the second sensitivity analysis and the five-clusters solution from the second sensitivity analysis. For each solution, trajectories were assigned to a cluster and weighted spaghetti plots of trajectories were created to visualise the cluster solutions (Figure S1). The median or mean trajectory is provided for solutions of a k-medoids algorithm or KmL algorithm respectively. All four-cluster solutions could be described with the clusters A = “low or no pain”, B = “mild pain”, C = “moderate pain”, D = “severe pain”. The five-cluster solution could be described with the same descriptors and E = “very severe pain”.

Figure S2: Weighted spaghetti plots of trajectories assigned to each cluster in (A) main analysis, (B) first sensitivity analysis, (C) four-cluster solution of second sensitivity analysis, (D) five-cluster solution of second sensitivity analysis. All cluster solutions can be described by A = “low or no pain”, B = “mild pain”, C = “moderate pain”, D = “severe pain”, with (D) also containing E = “very severe pain”.

The percentage of trajectories assigned to each cluster are shown in Table S1. Compared to the main analysis, the first sensitivity analysis had a larger percentage of trajectories in Cluster A, and a lower

Table S1: Percentage of trajectories assigned to each cluster in most optimal cluster solutions

| Cluster | Main analysis (%) | First sensitivity analysis (%) | Second sensitivity analysis (four-cluster solution) (%) | Second sensitivity analysis (five-cluster solution) (%) |
| --- | --- | --- | --- | --- |
| Cluster A (low/no pain) | 7.8 | 11.4 | 8.4 | 8.4 |
| Cluster B (mild) | 37.6 | 38.7 | 37.4 | 37.3 |
| Cluster C (moderate) | 38.2 | 36.5 | 40.9 | 38.8 |
| Cluster D (severe) | 16.3 | 13.4 | 13.3 | 11.0 |
| Cluster E (very severe) | NA | NA | NA | 4.5 |

percentage in Cluster D. Compared to the main analysis, the second sensitivity analysis had a smaller percentage of trajectories in Cluster D, in both the four- and five-cluster solutions. In the five-cluster solution, 4.5% of trajectories were assigned to Cluster E.

## Comparison of trajectory assignment

Each trajectory is assigned to a cluster in the four-cluster solutions of the main analysis, first sensitivity analysis and second sensitivity analysis. For the trajectories assigned to each cluster in the main analysis, Table S2 compares the percentage of trajectories assigned to the same cluster in the first sensitivity analysis and the second sensitivity analysis. Of the trajectories assigned to cluster A in the main analysis, at least 97% of trajectories are also assigned to Cluster A in both sensitivity analyses. However, of the trajectories assigned to cluster D in the main analysis, only 80.3% and 77.4% are still assigned to Cluster D in the first sensitivity analysis and second sensitivity analysis respectively. This suggests that a large proportion of trajectories are assigned to the same cluster in each of the analyses, but a significant proportion may be assigned to different clusters, perhaps reflecting the changes in cluster sizes among the different analyses.

| **Table S2:** Comparison of trajectories assigned to the same cluster as the main analysis | | |
| --- | --- | --- |
| Cluster in main analysis | Number (percentage) same in first sensitivity analysis | Number (percentage) same in second sensitivity analysis |
| Cluster A (low/no pain) | 1672 (97.5%) | 1714 (100%) |
| Cluster B (mild) | 7238 (87.8%) | 7822 (94.9%) |
| Cluster C (moderate) | 7108 (84.9%) | 7886 (94.1%) |
| Cluster D (severe) | 2877 (80.3%) | 2775 (77.4%) |
