# Supplementary material for: Identifying Weekly Trajectories of Pain Severity Using Daily Data From an mHealth Study: Cluster Analysis
Source: JMIR Mhealth Uhealth. 2024 Jul 19;12:e48582. doi: 10.2196/48582 (PMC11297369; doi:10.2196/48582)
Supplement: Multimedia Appendix 3 [file mhealth_v12i1e48582_app3.docx]

This section reports the results from the final sensitivity analysis. The main analysis defined complete participant weeks for each individual as those containing complete pain severity data for each day in a Monday-Sunday week. Six alternative definitions are explored here, using complete pain severity data for each day in (1) Tuesday-Monday weeks, (2) Wednesday-Tuesday weeks, (3) Thursday-Wednesday weeks, (4) Friday-Thursday weeks, (5) Saturday-Friday weeks and (6) Sunday-Saturday weeks. For each definition, all complete participant weeks were identified, compared using the Manhattan distance and clustered using the CLARA program of the *k*-medoids algorithm. The remaining variability within clusters (within-cluster sum of squares) was calculated for *k* (number of clusters) between 1 and 20. The results are presented in Figure S1, for each data set separately. A four-cluster solution is optimal for each dataset. The percentage of trajectories assigned to each cluster are reported in Table S1. The graphs in Figure S1 and cluster sizes in Table S1 show similarity in the results for trajectories starting on different days of the week, suggesting that the main analysis is robust to the day of the week on which the trajectory began.


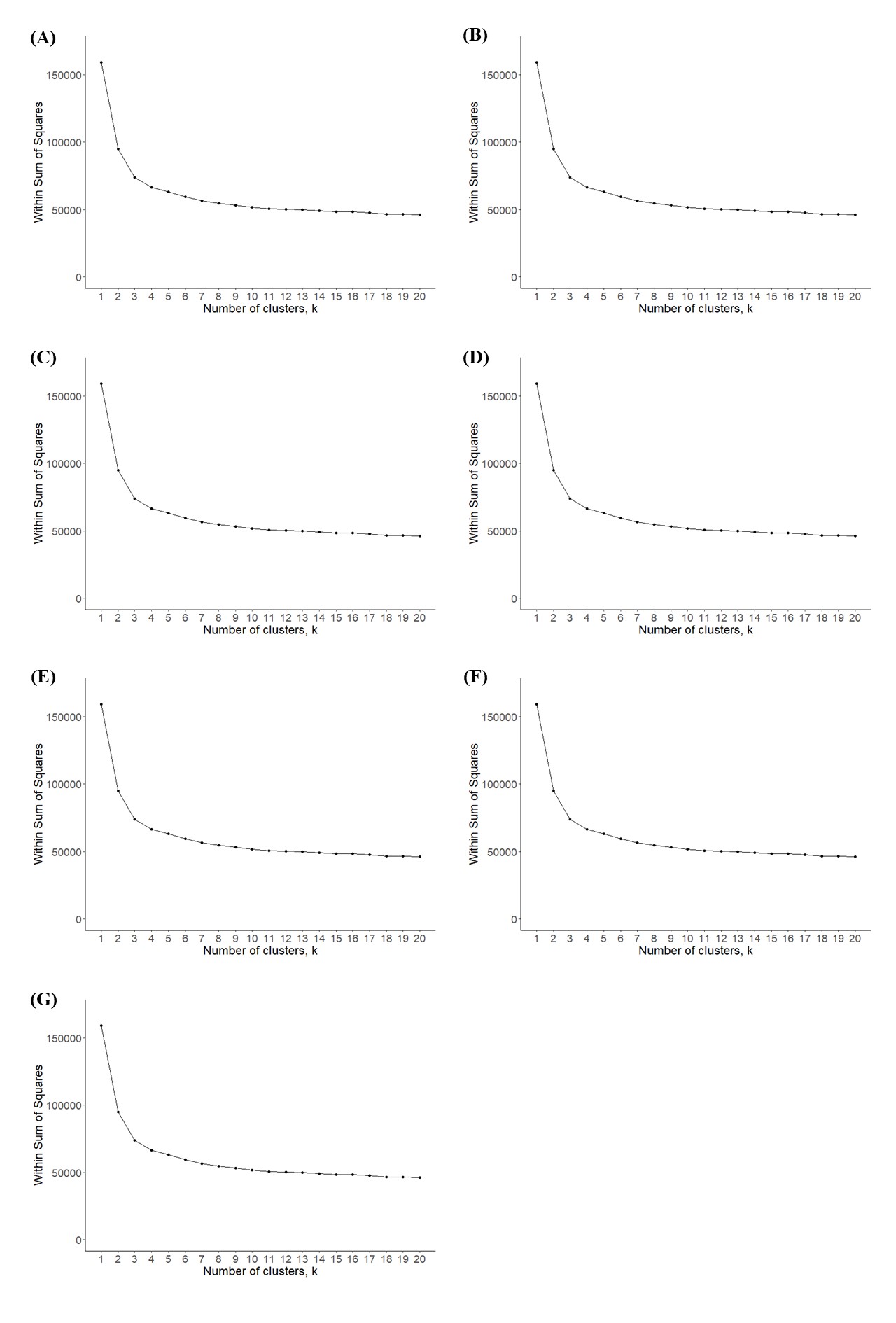


Figure S1: Unexplained variability among different cluster (k) solutions using trajectories with complete data (A) Monday – Sunday, (B) Tuesday – Monday, (C) Wednesday – Tuesday, (D) Thursday – Wednesday, (E) Friday – Thursday, (F) Saturday – Friday, (G) Sunday – Saturday.

| First day of trajectories | Monday | Tuesday | Wednesday | Thursday | Friday | Saturday | Sunday |
| --- | --- | --- | --- | --- | --- | --- | --- |
| Number of trajectories in analysis | 21919 | 22103 | 22320 | 22653 | 22404 | 22255 | 22067 |
| Percentage in Cluster A (low/no pain) | 7.8% | 7.8% | 7.9% | 7.8% | 7.9% | 7.7% | 7.7% |
| Percentage in Cluster B (mild pain) | 37.6% | 37.7% | 37.7% | 37.6% | 37.6% | 37.7% | 37.5% |
| Percentage in Cluster C (moderate pain) | 38.2% | 38.2% | 38.3% | 38.2% | 38.0% | 38.2% | 38.4% |
| Percentage in Cluster D (severe pain) | 16.3% | 16.3% | 16.2% | 16.4% | 16.4% | 16.4% | 16.3% |

Table S1: Comparison of cluster size among four-cluster solution for trajectories starting on different days of the week
