# Supplementary material for: Identifying Weekly Trajectories of Pain Severity Using Daily Data From an mHealth Study: Cluster Analysis
Source: JMIR Mhealth Uhealth. 2024 Jul 19;12:e48582. doi: 10.2196/48582 (PMC11297369; doi:10.2196/48582)
Supplement: Multimedia Appendix 4 [file mhealth_v12i1e48582_app4.docx]

Table S1: Comparison of demographics among participants included in the main analysis and participants included in the transition analysis.

|  |  | Number (percentage) of participants in main analysis | Number (percentage) of participants in transition analysis |
| --- | --- | --- | --- |
| Age | 1­7–24 | 67 (2.4) | 30 (1.7) |
|  | 25–34 | 255 (9.1) | 139 (7.9) |
|  | 35–44 | 508 (18.1) | 296 (16.8) |
|  | 45–54 | 755 (26.9) | 468 (26.6) |
|  | 55–64 | 788 (28.1) | 528 (30.0) |
|  | 65–86 | 434 (15.5) | 300 (17.0) |
| Sex | Female | 2333 (83.1) | 1467 (83.3) |
|  | Male | 474 (16.9) | 294 (16.7) |
| Condition* | Rheumatoid arthritis | 548 (19.5) | 365 (20.7) |
|  | Osteoarthritis | 975 (34.7) | 644 (36.6) |
|  | Spondyloarthropathy | 254 (9.0) | 161 (9.1) |
|  | Gout | 96 (3.4) | 60 (3.4) |
|  | Unspecific arthritis | 1028 (36.6) | 676 (38.4) |
|  | Fibromyalgia | 718 (25.6) | 446 (25.3) |
|  | Chronic headache | 274 (9.8) | 172 (9.8) |
|  | Neuropathic pain | 427 (15.2) | 253 (14.4) |
|  | Other/no medical diagnosis | 668 (23.8) | 402 (22.7) |

^a^Percentages exceed 100% because participants could report multiple chronic-pain conditions.
